# Increasing Membrane Cholesterol Level Increases the Amyloidogenic Peptide by Enhancing the Expression of Phospholipase C

**DOI:** 10.1155/2013/407903

**Published:** 2013-03-07

**Authors:** Yoon Sun Chun, Hyun Geun Oh, Myoung Kyu Park, Tae-Wan Kim, Sungkwon Chung

**Affiliations:** ^1^Department of Physiology, Samsung Biomedical Research Institute, Sungkyunkwan University School of Medicine, Suwon 440-746, Republic of Korea; ^2^Department of Pathology, Columbia University Medical Center, New York, NY 10032, USA

## Abstract

Cerebral elevation of 42-residue amyloid *β*-peptide (A*β*42) triggers neuronal dysfunction in Alzheimer's disease (AD). Even though a number of cholesterol modulating agents have been shown to affect A*β* generation, the role of cholesterol in the pathogenesis of AD is not clear yet. Recently, we have shown that increased membrane cholesterol levels downregulates phosphatidylinositol 4,5-bisphosphate (PIP_2_) via activation of phospholipase C (PLC). In this study, we tested whether membrane cholesterol levels may affect the A*β*42 production via changing PIP_2_ levels. Increasing membrane cholesterol levels decreased PIP_2_ and increased secreted A*β*42. Supplying PIP_2_, by using a PIP_2_-carrier system, blocked the effect of cholesterol on A*β*42. We also found that cholesterol increased the expressions of *β*1 and *β*3 PLC isoforms (PLC*β*1, PLC*β*3). Silencing the expression of PLC*β*1 prevented the effects of cholesterol on PIP_2_ levels as well as on A*β*42 production, suggesting that increased membrane cholesterol levels increased secreted A*β*42 by downregulating PIP_2_ via enhancing the expression of PLC*β*1. Thus, cholesterol metabolism may be linked to A*β*42 levels via PLC*β*1 expression and subsequent changes in PIP_2_ metabolism.

## 1. Introduction

AD is a progressive and irreversible neurodegenerative disorder leading to cognitive, memory, and behavioral impairments. Cerebral elevation and accumulation of A*β* are necessary steps in the pathogenesis of AD [1–3]. Sequential proteolytic cleavages of amyloid precursor protein (APP) by membrane-bound *β*-secretase and *γ*-secretase produce two major isoforms of A*β*, A*β*40, and A*β*42. Therefore, this pathway is called amyloidogenic pathway. More amyloidogenic A*β*42 is considered as a pathogenic agent [4, 5]. Alternatively, APP can be sequentially processed by *α*-secretase, and *γ*-secretase, precluding A*β* production (nonamyloidogenic pathway). Even though advanced age serves as a major risk factor, approximately 5% of AD cases are familial (FAD), and some of them are attributable to autosomal dominant mutations in presenilin (PS) genes, PS1 and PS2. PS1 and PS2 function as catalytic subunits of *γ*-secretase, and FAD mutations in PSs affect APP processing increasing the ratio of A*β*42 to A*β*40 [6–8].

Growing evidence indicates that dysregulation of lipid pathways have regulatory consequences for APP processing and A*β* generation [9]. Especially, cholesterol has been suggested to participate in the etiology of AD by increasing the generation of A*β* [10]. Cholesterol can directly regulate the activities of *β*-secretase or *γ*-secretase to alter amyloidogenesis [11–13]. Alternatively, changes in cholesterol level may affect the lipid environment for APP processing and A*β* generation. APP is located either within or outside of lipid rafts. Since BACE1 (*β*-secretase) is predominantly located in lipid rafts, APP processing occurring within lipid rafts is amyloidogenic, whereas APP processing occurring outside lipid rafts is considered nonamyloidogenic [14]. When cholesterol is depleted, the association of BACE1 with lipid rafts is decreased, producing less A*β* [15–17]. In contrast, increasing cholesterol induces the co-clustering of APP and BACE1, producing more A*β* [18]. From these results it could be hypothesized that high cholesterol levels may be responsible for initiating the pathogenesis of AD. However, it was recently demonstrated that lowering cholesterol levels results in increased *β*-amyloid production in neurons [19]. Abad-Rodriguez et al. reported that lowering the intracellular levels of cholesterol could increase the rate of amyloidogenic processing of APP by placing the hydrolyzing enzyme (BACE1) and APP in close proximity within the same intracellular compartments. Therefore, more experiments will be needed to clarify the conflicting results about the role of cholesterol in pathogenesis of AD.

PIP_2_ is known as one of phospholipid component of cell membrane [20], playing important regulatory roles in a variety of cell functions, such as rearrangement of the cytoskeleton and membrane trafficking [21]. We have reported that FAD-linked PS mutants down-regulate PIP_2_ levels, and that PIP_2_ levels are inversely correlated to the production of A*β*42 [22]. We also demonstrated that increased membrane cholesterol level decreases the level of PIP_2_ via the activation of PLC [23]. Therefore, there exists a crosstalk between two plasma membrane-enriched lipids, cholesterol and PIP_2_. Considering the close relationship between PIP_2_ levels and the production of A*β*42, we suspected that increased membrane cholesterol levels affect the A*β*42 production via down-regulating PIP_2_ levels. In this study, we found that membrane cholesterol decreased PIP_2_ levels and increased secreted A*β*42. Supplying PIP_2_ by using a PIP_2_-carrier system blocked the effect of cholesterol, which might indicate that the effect of cholesterol on A*β*42 was by downregulation of PIP_2_ levels. Enriching membrane with cholesterol increased the expression of some PLC isoforms, such as PLC*β*1 and PLC*β*3. Blocking the new protein synthesis prevented the effect of cholesterol on PIP_2_ levels as well as on A*β*42 production. We found that the expression of PLC*β*1 was specifically linked to A*β*42 production. These results suggest that increased membrane cholesterol levels, and FAD-linked PS mutations may share the same molecular mechanism, that is, the downregulation of PIP_2_, which may serve as the molecule linking cholesterol metabolism to the pathogenesis of AD.

## 2. Materials and Methods 

### 2.1. Cell Culture

 HeLa cells stably transfected with APP_751_ carrying the Swedish mutation (APPsw) were cultured at 37°C, 5% CO_2_, in Dulbecco's Modified Eagle Medium (DMEM) supplemented with 10% heat-inactivated fetal bovine serum containing 100 units/mL penicillin, 100 *μ*g/mL streptomycin, 260 *μ*g/mL Zeocin, and 400 *μ*g/mL G418. Human neuroblastoma SH-SY5Y cells were cultured in DMEM containing 10% heat-inactivated fetal bovine serum, 100 units/mL penicillin, and 100 *μ*g/mL streptomycin.

### 2.2. Procedure

To enrich the cells with cholesterol, cells were exposed to DMEM culture medium containing methyl-*β*-cyclodextrin (M*β*CD, Sigma, USA) saturated with cholesterol (water-soluble cholesterol). During the incubation, cells were maintained in a humidified CO_2_ incubator at 37°C. In some experiments, to avoid the use of M*β*CD, cells were incubated with cholesterol which was solubilized by sonication. For this purpose, cholesterol in methanol/chloroform mixture (1 : 1 v/v) was dried under nitrogen gas and sonicated for 2 min in phosphate-buffered saline before use.

In some experiments, cells were pretreated with 10 *μ*M Actinomycin-D (Sigma) or 50 *μ*g/mL cyclohexamide (Sigma) for 0.5 h before 75 *μ*M water-soluble cholesterol was added. PIP_2_ were delivered into the cells using the PIP_2_-carrier system (Echelon Bioscience Inc., USA). Carrier-PIP_2_ complex was incubated with APP-transfected HeLa cells for 4 h in the absence or presence of 75 *μ*M water-soluble cholesterol.

### 2.3. Antisense Oligonucleotides Treatments

The antisense oligonucleotides (IDT, USA) targeted at PLC*β*1 and PLC*β*3 were designed to be complementary to the 5′ sequences and were phosphorothionated at all positions to minimize intracellular cleavage by enzymes and to enhance their stability (5′-actccgggttgagccccggc-3′ for PLC*β*1 and 5′-tccaactgcagcgcgtggac-3′ for PLC*β*3). Antisense oligonucleotide (5′-gccccgtatgaccgcgccgg-3′) having no target was used as a control in all of experiments. The APP-transfected HeLa cells were plated at a density of 2 × 10^6^ cells per 60 mm dish and incubated overnight and then treated with the 10 *μ*M antisense oligonucleotides for 4 h in DMEM culture medium without serum. After treatment, the medium was replaced by a new medium containing 10 *μ*M antisense oligonucleotides with or without water-soluble cholesterol for 2 h. Media were collected to measure levels of A*β*, and cells were homogenized to confirm PLC expression levels and PIP_2_ levels. 

### 2.4. Cholesterol Assay

Filipin staining of cells (0.05%, DMSO 1%) was performed for 1 h at room temperature after cholesterol enrichment to confirm the changes of free cholesterol levels at the plasma membrane. Fluorescence images were obtained using a LSM 710 confocal microscope (Zeiss) using laser emitting at 351 nm. Images were quantified to obtain the mean fluorescence density values of plasma membrane from the edge of the cell to 500 nm inside using the ImageJ program.

### 2.5. sAPP*α*, sAPP*β*, and A*β* Peptide Assay

Levels of A*β* peptides were assayed by using the Invitrogen. A*β* ELISA kits (USA) or Wako *β*-amyloid ELISA kits (high-sensitive; Japan). For sAPP*α*, sAPP*β* detection, samples were analyzed by ELISA kit from IBL (USA). APP-transfected HeLa cells at 80% confluence in a 35 mm dish were cultured for 8 h with water-soluble cholesterol in DMEM culture medium without serum. Control cells were treated similarly and incubated with serum-free DMEM solution without any cholesterol. After exposure to cholesterol, supernatants were collected to measure levels of A*β*, sAPP*α*, or sAPP*β*. To detect A*β* from SH-SY5Y cells, supernatants were desalted using PD-10 desalting column (GE Healthcare, USA), dried, and reconstituted in water. The samples were analyzed by ELISA kits according to the supplier's instructions.

### 2.6. Protein Extraction

Cell fractionations were obtained by homogenizing with hypotonic buffer using a 23-gauge needle. The samples were then centrifuged at 1,000 ×g for 10 min at 4°C to remove nuclei and debris. Supernatants were separated by centrifugation at 100,000 ×g for 1 h at 4°C into membrane (pellet) and cytosol (supernatant) fractions. Whole cell lysates were prepared by homogenizing with lysis buffer (10 mM Tris-HCl, 150 mM NaCl, 1% Triton X-100, 0.25% Nonidet P-40, 2 mM EDTA, pH7.4) using a cell scraper. The lysed cells were centrifuged at 12,000 ×g for 10 min at 4°C. The protein in the supernatant was determined by Bradford assay (Bio-rad, USA).

### 2.7. Western Blot Analysis

Proteins were resolved on SDS-PAGE and transferred to nitorcelluose membrane. Membranes were blocked with 5% nonfat milk powder in Tris-buffered saline/Tween 20 (TBST) for 1 h at room temperature, then incubated with rabbit polyclonal anti-PLC*β*1 (SC-9050), PLC*β*2 (SC-206), PLC*β*3 (SC-13958), PLC*β*4 (SC-20760), PLC*γ*2 (SC-9015), mouse monoclonal anti-PLC*γ*1 (SC-7290) antibodies (Santa Cruz Biotechnology, USA), anti-APP antibody (LN27, Zymed), anti *β*-actin (A5441, Sigma), and rabbit anti *β*-tubulin (T2200, Sigma) for overnight at 4°C. Dilutions were 1 : 500 for PLC isozymes and 1 : 4000 for *β*-tubulin, *β*-actin, and APP. After washing, membranes were incubated for 1 h at room temperature with horseradish peroxidase-conjugated goat anti-rabbit IgG or goat anti-mouse IgG antibodies (1 : 2000 dilution; Zymed, USA) and washed. Peroxidase activity was visualized with enhanced chemilluminescence. Blots were quantified with the Multi Gauge software using a LAS-3000 system (FugiFilm, Japan).

### 2.8. PIP_2_ Assay

The amount of PIP_2_ extracted from APP-transfected HeLa cells were measured by using PIP_2_ Mass ELISA kit (Echelon Biosciences Inc., USA). PIP_2_ was extracted from the control cells or cells treated with water-soluble cholesterol according to the supplier's instructions. Cellular PIP_2_ quantities were estimated by comparing the values from the standard curve, which showed linear relationship at the range from 0.5 to 1000 pM concentrations. 

### 2.9. Statistical Analysis

Data was expressed as mean ± SEM. Statistical comparisons between the controls and treated experimental groups were performed using the Student's *t*-test. *P* < 0.05 was considered statistically significant.

## 3. Results

### 3.1. Increasing Membrane Cholesterol Levels Downregulates PIP_2_ and Increases Secreted A*β*42

**Figure 1:**
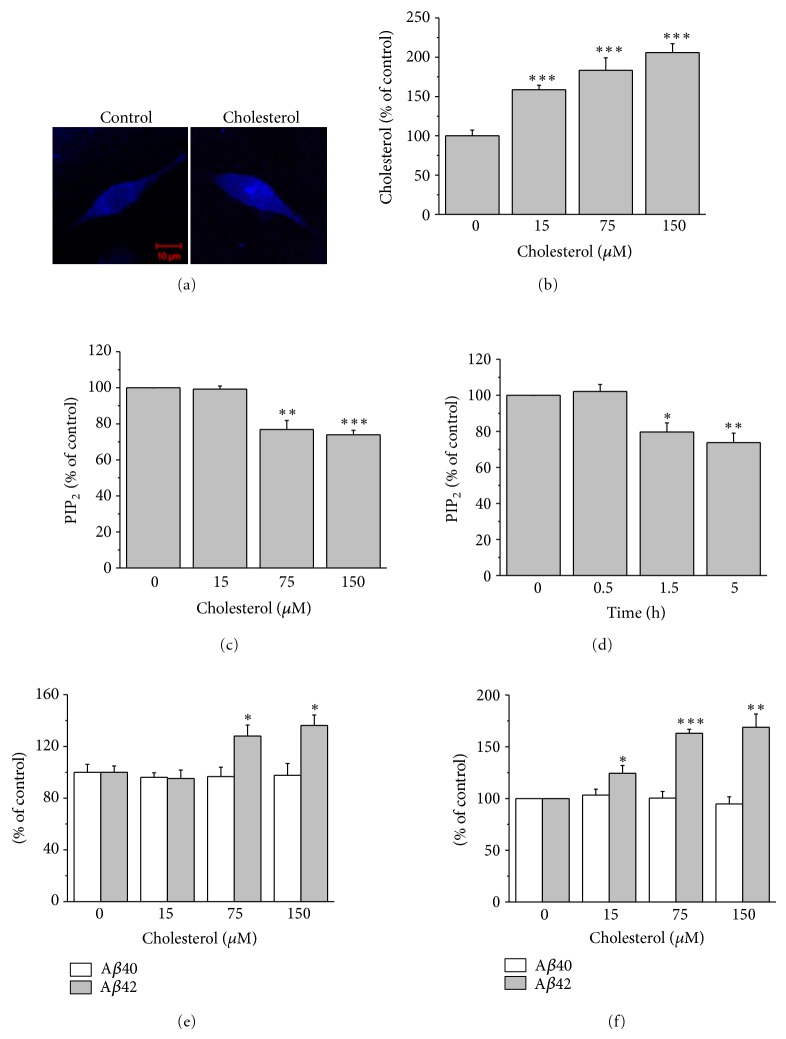
Augmentation of membrane cholesterol levels downregulated PIP_2_ levels and increased A*β*42. (a, b) Incubating APP-transfected HeLa cells with water-soluble cholesterol increased cholesterol levels in the plasma membrane. Cells were incubated with 0, 15, 75, and 150 *μ*M water-soluble cholesterol for 8 h at 37°C. Filipin staining was performed for 1 h at room temperature after cholesterol enrichment. (a) A typical fluorescence image with 75 *μ*M water-soluble cholesterol is shown in (b). Incubating the cells with water-soluble cholesterol increased the cholesterol contents in a concentration-dependent manner. Fluorescent intensities from plasma membranes were quantified as described in Section 2 (*n* = 6). (c) Incubating cells with water-soluble cholesterol downregulated PIP_2_ levels. APP-transfected HeLa cells were incubated for 8 h with 0, 15, 75, and 150 *μ*M water-soluble cholesterol. PIP_2_ levels in the membrane fractions were measured by using a PIP_2_ ELISA kit as described in Section 2 (*n* = 6). (d) Incubating cells with water-soluble cholesterol downregulated PIP_2_ levels in time-dependent manner. Cells were incubated with 75 *μ*M water-soluble cholesterol for 0.5, 1.5, and 5 h (*n* = 6). (e) Incubating cells with water-soluble cholesterol selectively increased secreted A*β*42 levels (closed bars; *n* = 6). In contrast, the levels of A*β*40 were not changed by cholesterol enrichment (open bars; *n* = 6). APP-transfected HeLa cells were incubated with 0, 15, 75, and 150 *μ*M water-soluble cholesterol for 8 h. A*β*40 and A*β*42 levels were measured from the conditioned media by using ELISA method as described in Section 2. (f) Incubating cells with water-soluble cholesterol increased secreted A*β*42 levels (closed bars; *n* = 6) but not A*β*40 levels (open bars; *n* = 4) from neuroblastoma SH-SY5Y cells. The endogenous A*β*40 and A*β*42 levels were measured from the conditioned media by using ELISA method. ∗*P* < 0.05; ∗∗*P* < 0.01; ∗∗∗*P* < 0.001.

M*β*CD, a water-soluble cyclic oligosaccharide, has hydrophobic cavity that is able to encapsulate insoluble compounds, thus enhances the solubility of cholesterol. M*β*CD saturated with cholesterol (water-soluble cholesterol) has been used to increase membrane cholesterol level, since it acts as a cholesterol donor [24–26]. We incubated APP-transfected HeLa cells with 15, 75, or 150 *μ*M water-soluble cholesterol for 8 h, and filipin staining was performed for 1 h at room temperature to monitor the membrane cholesterol level. Typical confocal image in Figure 1(a) shows that the membrane cholesterol level increased by 75 *μ*M water-soluble cholesterol. The changes of cholesterol level were confirmed by quantifying the filipin fluorescent intensities from plasma membranes. By incubating cells with 15 and 75 *μ*M water-soluble cholesterol, the fluorescent intensities were increased by 58.5 ± 5.8% and 83.3 ± 15.9% (*n* = 6), respectively (Figure 1(b)). We also tested the time-dependent accumulation of cholesterol in the membrane by incubating cells with 75 *μ*M water-soluble cholesterol. Cholesterol levels increased after 0.5 h, and it steadily increased further after 1.5 h or 5 h (Supplementary Figures  1(a) and 1(b), see Supplementary Material available online at http://dx.doi.org/10.1155/2013/407903). From these results we concluded that the direct administration of the water-soluble cholesterol leads to the increases in the membrane cholesterol levels. 

Recently, we have reported that augmentation of membrane cholesterol levels downregulates PIP_2_ level [23]. To validate this observation in the current system, APP-transfected HeLa cells were incubated with 15, 75, or 150 *μ*M water-soluble cholesterol for 8 h, and the steady state levels of PIP_2_ were measured using a PIP_2_ ELISA. PIP_2_ levels in 75 and 150 *μ*M cholesterol-treated cells were downregulated by 23.2 ± 5.0% and 26.1 ± 2.5% (*n* = 6), respectively (Figure 1(c)). We also tested the time-dependent effect of increased membrane cholesterol on the levels of PIP_2_ by incubating cells with 75 *μ*M water-soluble cholesterol. As shown in Figure 1(d), the steady state levels of PIP_2_ after 1.5 h and 5 h incubation time were downregulated by 20.3 ± 5.1% and 26.3 ± 5.3% (*n* = 6), respectively.

Since we have reported that cellular PIP_2_ levels are closely correlated with the A*β*42 levels [22], we tested the effect of increased membrane cholesterol levels on secreted A*β*. For this purpose, APP-transfected HeLa cells were incubated for 8 h with 15, 75, or 150 *μ*M water-soluble cholesterol, and A*β* levels were measured from the conditioned media by using an ELISA kits specific for A*β*40 or A*β*42. The secreted A*β*40 levels were not changed by increased membrane cholesterol levels (open bars in Figure 1(e)). However, A*β*42 levels were increased by 28.1 ± 8.4%, and 36.2 ± 8.1% (*n* = 6) when cells were incubated with 75, and 150 *μ*M water-soluble cholesterol, respectively (closed bars in Figure 1(e)). We also tested the effect of increased membrane cholesterol levels on the levels of secreted A*β* from neuroblastoma SH-SY5Y cells. The level of endogenous A*β*42 increased significantly (closed bars in Figure 1(f)), while the endogenous A*β*40 level was not changed (open bars in Figure 1(f)). Thus, these results suggest that the effect of cholesterol enrichment is specific to A*β*42 levels, and is not cell-type specific.

**Figure 2:**
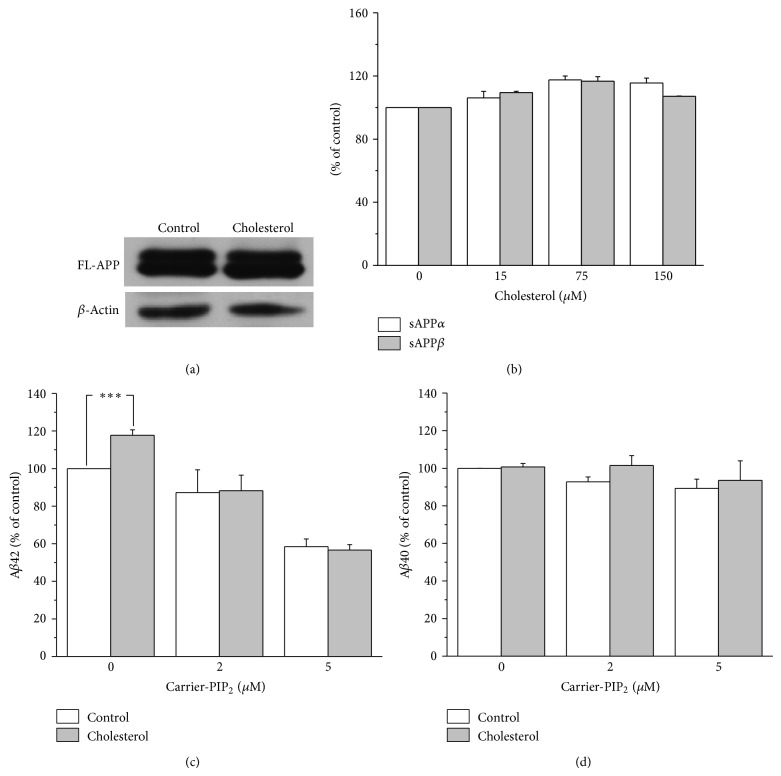
Elevation of PIP_2_ level prevented the effect of cholesterol on A*β*42 production. (a) Incubating APP-transfected HeLa cells with water-soluble cholesterol did not significantly change the full-length APP level. Cells were incubated with 75 *μ*M water-soluble cholesterol for 8 h. Similar Western blotting results were obtained from 4 different experiments. (b) Both sAPP*α* and sAPP*β* were slightly increased by incubating cells with 75 *μ*M water-soluble cholesterol for 8 h (*n* = 4). The levels of sAPP*α* and sAPP*β* were measured from the conditioned media as described in Section 2. (c) Supplying PIP_2_ decreased A*β*42 production and prevented the effect of cholesterol. APP-transfected HeLa cells were incubated with 0, 2, and 5 *μ*M carrier-PIP_2_ complex in the absence and the presence of 75 *μ*M water-soluble cholesterol for 4 h. A*β*42 levels were measured from the conditioned media by using ELISA method. Without treating cells with water-soluble cholesterol, A*β*42 production decreased as PIP_2_ concentration increased (*n* = 6, open bars). As expected, treating cells with water-soluble cholesterol increased the production A*β*42 in the absence of PIP_2_ (the first closed bar). However, the effects of cholesterol on A*β*42 were prevented by the presence of 2 *μ*M and 5 *μ*M PIP_2_ (the second and the third close bars, *n* = 6). (d) Elevation of PIP_2_ level did not change A*β*40 production (*n* = 5). ∗∗∗*P* < 0.001.

A*β* is produced by the sequential cleavages of APP by *β*-secretase followed by *γ*-secretase. Alternatively, APP can be cleaved sequentially by *α*-secretase followed by *γ*-secretase precluding the production of A*β*. Thus, the effect of increased membrane cholesterol levels on A*β* levels can occur in any of those processes. To begin to investigate the effects of membrane cholesterol on APP processing, we first examined the levels of full-length APP. Increased membrane cholesterol levels led to a moderate increase in the full-length APP levels (Figure 2(a)). However, the increase was less than 10% (*n* = 4). Then, we tested the effects of increased membrane cholesterol levels on the activities of *α*-secretase and *β*-secretase. For this purpose, we measured the levels of sAPP*α* and sAPP*β* from the conditioned media using specific ELISA kits, since they are produced via the activities of *α*-secretase and *β*-secretase, respectively. In this experiment, we used sAPP*β* ELISA kit for Swedish mutant. As shown in Figure 2(b), the levels of both sAPP*α* and sAPP*β* were also slightly increased by increased membrane cholesterol levels, which might be due to the increased level of their precursor, APP. However, the amount of increased sAPP*β* level was not robust to explain the A*β*42-selective changes associated with increased membrane cholesterol levels. Since membrane cholesterol levels affect A*β*42 but not A*β*40, it is conceivable that the effects of cholesterol may influence the specificity of *γ*-secretase-mediated cleavage of amyloidogenic APP C-terminal fragments (e.g., C99).

### 3.2. Intracellular Delivery of PIP_2_ Prevents the Effect of Increased Membrane Cholesterol Levels on Secreted A*β*42

In order to elucidate the role of PIP_2_ for the effect of increased membrane cholesterol levels on secreted A*β*42, we used a PIP_2_-carrier system for the intracellular delivery of PIP_2_. Because carrier compounds are “charge-neutralization” species, it could deliver the anionic PIP_2_ into the cells [27]. After the carriers were added at a one-to-one molar ratio with PIP_2_ at room temperature, the complex was diluted to the desired final concentration. Then, the carrier-PIP_2_ complex was incubated with cells for 4 h before A*β* levels were measured from the conditioned media.

The presence of 2 *μ*M and 5 *μ*M carrier-PIP_2_ complex decreased secreted A*β*42 levels by 12.8 ± 12.1% and 41.5 ± 4.1% (*n* = 6), respectively (open bars in Figure 2(c)). This result is consistent with our previous result showing the close correlation between PIP_2_ levels and A*β*42 production [22]. In the absence of carrier-PIP_2_ complex, incubating cells with 75 *μ*M water-soluble cholesterol for 4 h increased A*β*42 levels by 17.7 ± 2.9% (*n* = 6), which is consistent with the result in Figure 1(e). However, the presence of either 2 *μ*M or 5 *μ*M carrier-PIP_2_ complex completely prevented the effect of water-soluble cholesterol on A*β*42 levels (closed bars in Figure 2(c)). These results suggest that the relative levels of cholesterol and PIP_2_ correlate closely with secreted A*β*42 levels in a positive or negative manner, respectively. Unlike A*β*42, the A*β*40 levels were not affected by the presence of carrier-PIP_2_ complex (open bars in Figure 2(d)). Also, the A*β*40 levels were not affected by increased membrane cholesterol levels in the presence of carrier-PIP_2_ complex (closed bars in Figure 2(d)), which was consistent with the specific effect of cholesterol on A*β*42 level.

### 3.3. Increasing Membrane Cholesterol Level Increases PLC*β*1 and PLC*β*3 Expressions

**Figure 3:**
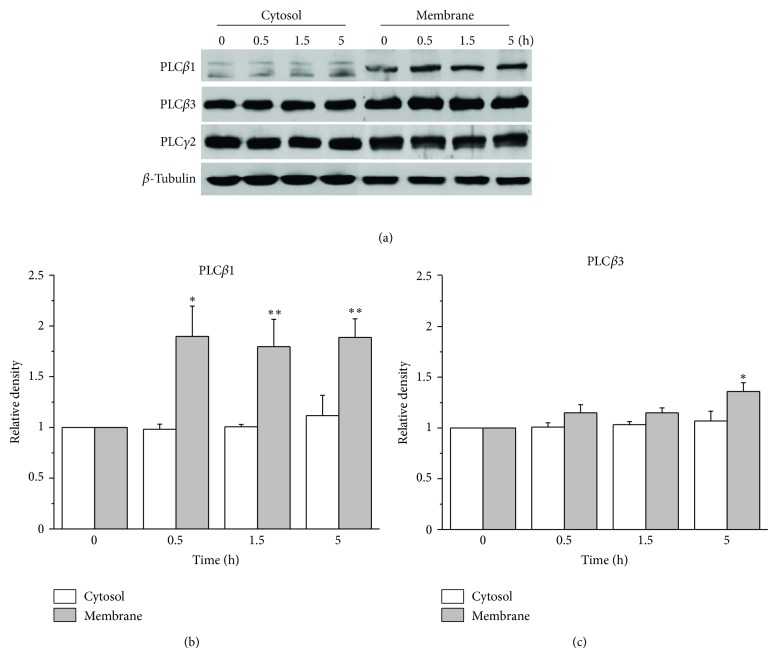
Augmentation of membrane cholesterol levels increased PLC*β*1 and PLC*β*3 expressions. (a) Representative western blotting results showed specific increase of PLC*β*1 and PLC*β*3 expressions from APP-transfected HeLa cells. Cells were incubated with 75 *μ*M water-soluble cholesterol for the indicated times. Membrane and cytosol fractions were obtained as described in Materials and Methods. Similar results were obtained from 5 different experiments. Note that PLC*β*1 and PLC*β*3 expressions were increased in time-dependent manner by cholesterol from membrane fraction but not from cytosol fraction. In contrast, PLC*γ*2 expression was not changed by cholesterol. *β*-tubulin was used to confirm the amount of proteins loaded. (b, c) Bars correspond to the densitometric analysis of PLC*β*1 and PLC*β*3 expressions from membrane and cytosol fractions (*n* = 5). ∗*P* < 0.05; ∗∗*P* < 0.01.

The major catabolic pathway for PIP_2_ is the hydrolysis by membranous PLC. Since we suspected that the effect of increased membrane cholesterol levels on the secreted A*β*42 levels is due to the downregulation of PIP_2_, we tested whether increased membrane cholesterol levels affect the activity of PLC. We first examined the expression levels of PLC isoforms by monitoring them using Western blot analysis from cytosol and membrane fractions in APP-transfected HeLa cells. The expression levels of PLC*β*1 were significantly increased by cholesterol only in membrane fractions as shown in Figure 3(a). Densitometry analysis of the bands corresponding to PLC*β*1 clearly supports this conclusion (Figure 3(b); *n* = 5). After 0.5 h incubation time with 75 *μ*M water-soluble cholesterol, expression levels of PLC*β*1 were increased by two folds in membrane fractions. The effect of cholesterol on PLC*β*1 expression lasted as long as 5 h. The expression level of PLC*β*3 was also increased in membrane fractions by cholesterol (Figures 3(a) and 3(c)). However, the effect was significant only after 5 h incubation time.

We used M*β*CD to increase cholesterol levels. However, the use of M*β*CD may cause nonspecific effects in addition to enrichment of membrane cholesterol levels [28]. To avoid the use of M*β*CD, free cholesterol was solubilized in phosphate-buffered saline using sonication. When cells were incubated with 75 *μ*M solubilized cholesterol for 1 h, the expression of PLC*β*1 in the membrane fraction was increased (Supplementary Figure  2). At 1 h incubation time, the expression of PLC*β*3 was not changed. These results indicated that the effect of cholesterol on PLC*β*1 expression was not due to the nonspecific effect of M*β*CD.

Cholesterol-induced increases in the levels of PLC*β*1 and PLC*β*3 were observed almost exclusively in the membrane fraction. Also, increased membrane cholesterol levels did not change the expression levels of PLC*γ*2 as shown in Figure 3(a) from a typical experiment. The expression levels of other PLC isoforms (PLC*β*2, PLC*β*4, and PLC*γ*1) were not changed either (Supplementary Figure  3(a)). We also observed specific increase of PLC*β*1 and PLC*β*3 expressions from SH-SY5Y cells by enriching membrane cholesterol levels (Supplementary Figure  3(b)). 

**Figure 4:**
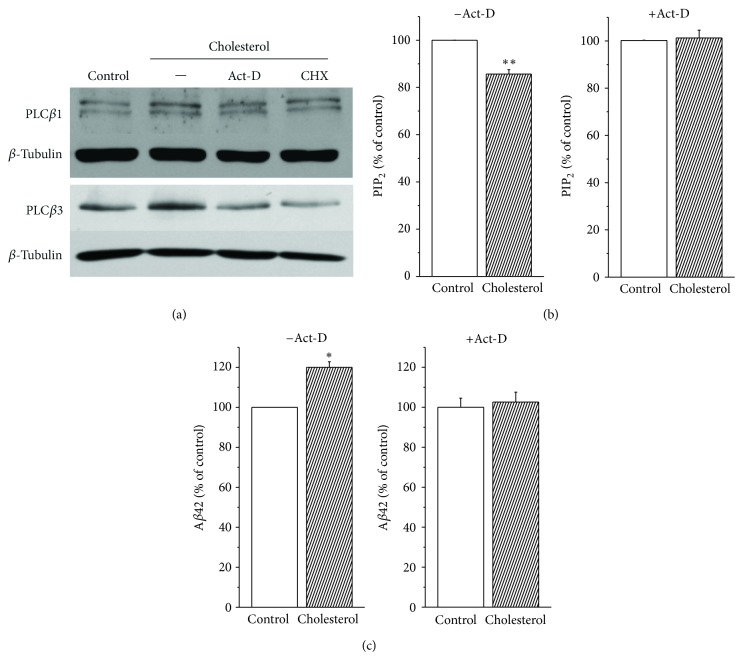
Transcription inhibitor prevented the effects of augmentation of membrane cholesterol levels. (a) Transcription and translation inhibitors prevented the effect of cholesterol on PLC*β*1 and PLC*β*3 expressions. APP-transfected HeLa cells were pretreated with the transcription inhibitor, actinomycin-D (Act-D, 10 *μ*M) or translation inhibitor, and cyclohexamide (CHX, 50 *μ*g/mL) for 10 min, which was followed by the additional 0.5 h incubation with 75 *μ*M water-soluble cholesterol. In the presence of Act-D or CHX, the effect of cholesterol on PLC*β*1 and PLC*β*3 expressions in membrane fractions was prevented. Similar results were obtained from 3 different experiments. *β*-tubulin was used to confirm the amount of proteins loaded. (b) Transcription inhibitor prevented the effect of cholesterol on PIP_2_ levels. APP-transfected HeLa cells were incubated in the presence or absence of 10 *μ*M Act-D with 75 *μ*M water-soluble cholesterol for 1 h. PIP_2_ levels in membrane fractions were measured by using a PIP_2_ ELISA kit as described in Section 2. In the absence of Act-D (−Act-D), cholesterol decreased PIP_2_ levels (*n* = 6). However, the presence of Act-D (+Act-D) prevented the effect of cholesterol on PIP_2_ levels (*n* = 6). (c) Transcription inhibitor prevented the effects of cholesterol on A*β*42 production. APP-transfected HeLa cells were incubated in the presence or absence of 10 *μ*M Act-D with 75 *μ*M water-soluble cholesterol for 4 h. A*β*42 levels were measured from the conditioned media by using ELISA method. In the absence of Act-D (−Act-D), cholesterol increased A*β*42 level (*n* = 4). However, the presence of Act-D (+Act-D) prevented the increase of A*β*42 production induced by cholesterol (*n* = 4). ∗*P* < 0.05; ∗∗*P* < 0.01.

To determine whether the effect of cholesterol on PLC expression was due to increased transcription, cells were preincubated for 10 min with the transcription inhibitor, actinomycin-D (Act-D, 10 *μ*M), or with the translation inhibitor, cyclohexamide (CHX, 50 *μ*g/mL). Then, cells were incubated further for 0.5 h with 75 *μ*M water-soluble cholesterol. Representative Western blots for PLC*β*1 and PLC*β*3 from membrane fractions are shown in Figure 4(a). The effect of cholesterol on PLC*β*1 and PLC*β*3 expressions was completely prevented either by Act-D or by CHX, indicating that the effect of cholesterol on PLC*β*1 and PLC*β*3 expression was via the up-regulation of transcription. Similar results were obtained from 4 different experiments. Then, we tested the effect of Act-D on the steady state level of PIP_2_. As we expected, PIP_2_ levels were downregulated by 18.9 ± 0.3% (*n* = 6) when cells were treated with 75 *μ*M water-soluble cholesterol for 1 h in the absence of Act-D (Figure 4(b)). However, the effect of cholesterol on PIP_2_ levels was completely prevented when cells were pretreated with Act-D (*n* = 6). These results confirmed that increased membrane cholesterol levels increases the expression of PLC*β*1 and PLC*β*3, leading to the downregulation of PIP_2_ levels. Next, we tested the effect of Act-D on the level of secreted A*β*42. For this purpose, cells were incubated for 4 h with 75 *μ*M water-soluble cholesterol with or without Act-D. As expected, cholesterol increased the levels of secreted A*β*42 by 20.1 ± 2.8% (*n* = 4) in the absence of Act-D (Figure 4(c)). However, the presence of Act-D completely prevented the effect of increased membrane cholesterol levels on A*β*42 (*n* = 4). These results suggest that the increase of PLC transcription by cholesterol induced the downregulation of PIP_2_ levels, which increased secreted A*β*42 levels.

### 3.4. Inhibition of PLC*β*1 Expression Prevents the Effect of Increased Membrane Cholesterol Levels on Secreted A*β*42

**Figure 5:**
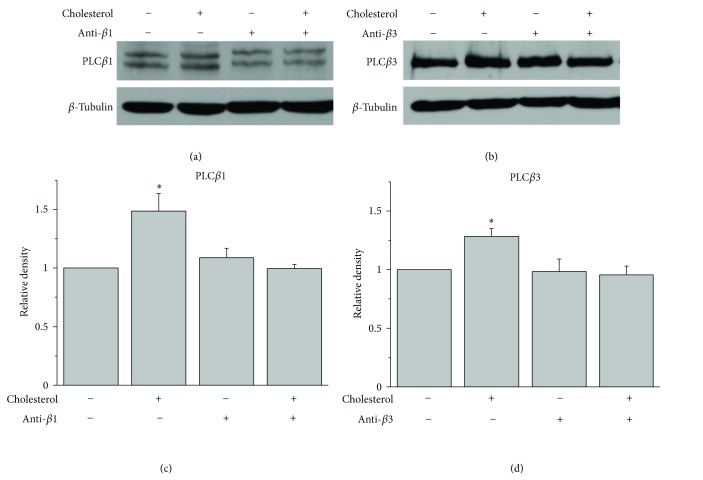
The effect of augmentation of membrane cholesterol levels on PLC*β*1 and PLC*β*3 expressions were blocked by the presence of antisense oligonucleotides. (a, b) The effect of cholesterol on PLC*β*1 and PLC*β*3 expressions were prevented by the presence of antisense oligonucleotides. APP-transfected HeLa cells were incubated with or without 75 *μ*M water-soluble cholesterol for 2 h, and the expression levels of PLC*β*1 and PLC*β*3 were tested using western blotting. In some cells, 10 *μ*M antisense oligonucleotides directed against PLC*β*1 (anti *β*1) or PLC*β*3 (anti *β*3) were pretreated for 4 h before the cholesterol treatment. Similar results were obtained from 4 different experiments. Antisense oligonucleotides having no specific target were used for controls in all of experiments. (c, d) Bars correspond to the densitometric analysis of the expression levels of PLC*β*1 and PLC*β*3, respectively (*n* = 6). ∗*P* < 0.05.

We tested whether PLC*β*1 or PLC*β*3 is required for the observed effects of cholesterol on A*β*42. To avoid any further transfection, cells were pretreated with 10 *μ*M antisense oligonucleotides against PLC isoforms for 4 h. Then the media were replaced by fresh media containing the same antisense oligonucleotides with or without 75 *μ*M water-soluble cholesterol, followed by additional incubation for 2 h. Antisense oligonucleotides having no specific target were used for controls in all of experiments. A typical western blot for PLC*β*1 is shown in Figure 5(a), and the band densities are expressed relative to *β*-tubulin density in Figure 5(c) (*n* = 6). As we expected, cholesterol increased PLC*β*1 expression. However, the presence of antisense oligonucleotides against PLC*β*1 (anti *β*1) blocked the effect of cholesterol. Similarly, an increase of PLC*β*3 expression by cholesterol was blocked by antisense oligonucleotides against PLC*β*3 (anti *β*3; Figures 5(b) and 5(d)). These results demonstrated that these antisense oligonucleotides were very effective to block the effect of increased membrane cholesterol levels on the expression of PLC*β*1 and PLC*β*3.

**Figure 6:**
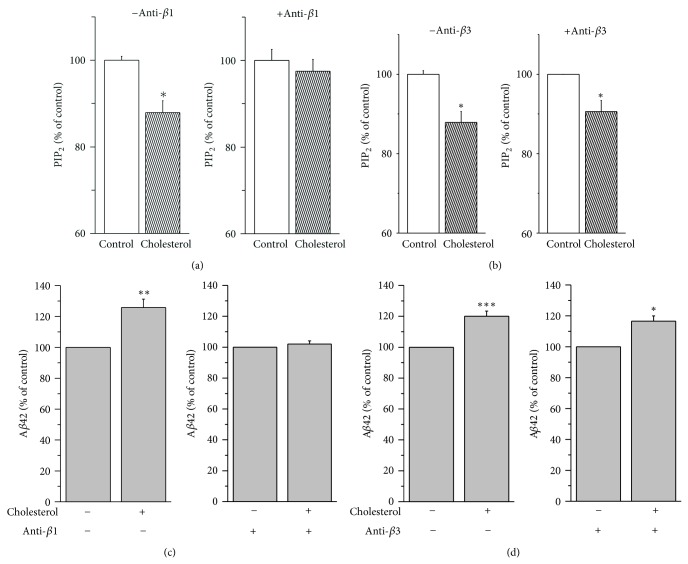
Suppression of PLC*β*1 expression prevented the effects of augmentation of membrane cholesterol levels on PIP_2_ levels and A*β*42 production. (a, b) The effect of cholesterol on PIP_2_ levels was blocked in the presence of antisense oligonucleotides directed against PLC*β*1. APP-transfected HeLa cells were incubated with or without 75 *μ*M water-soluble cholesterol for 2 h, and PIP_2_ levels in the membrane fractions were measured by using a PIP_2_ ELISA kit. In some cells, antisense oligonucleotides directed against PLC*β*1 or PLC*β*3 (10 *μ*M) were pretreated for 4 h before the cholesterol treatment. The presence of PLC*β*1 antisense oligonucleotides (*n* = 6), but not that of PLC*β*3, prevented the effects of cholesterol (*n* = 6). (c, d) The effect of cholesterol on A*β*42 production was blocked in the presence of antisense oligonucleotides directed against PLC*β*1. After treating cells as in (a) and (b), A*β*42 levels were measured from the conditioned media by using ELISA method. The presence of PLC*β*1 antisense oligonucleotides (*n* = 8), but not that of PLC*β*3, blocked the effects of cholesterol enrichment (*n* = 6). ∗*P* < 0.05; ∗∗*P* < 0.01; ∗∗∗*P* < 0.001.

Then, we tested the effect of these antisense oligonucleotides on the PIP_2_ levels. In the presence of control antisense oligonucleotides (−anti *β*1), cholesterol decreased PIP_2_ levels by 12.1 ± 2.8% (Figure 6(a); *n* = 6). In the presence of antisense oligonucleotide against PLC*β*1 (+anti *β*1), cholesterol decreased PIP_2_ levels only by 2.5 ± 1.0% (*n* = 6). In contrast, cholesterol decreased PIP_2_ levels by 10 ± 2.8% (*n* = 6) even in the presence of antisense oligonucleotide against PLC*β*3 (+anti *β*3; Figure 6(b)). Thus, inhibiting PLC*β*1 expression, but not inhibiting PLC*β*3 expression, prevented the effect of increased membrane cholesterol levels on PIP_2_ levels. These results may suggest that the downregulation of PIP_2_ levels by cholesterol enrichment is specifically via the increased expression of PLC*β*1.

Next, we tested the effect of increased membrane cholesterol levels on the levels of secreted A*β*42 in the presence of these antisense oligonucleotides. As we expected, cholesterol increased the levels of secreted A*β*42 by 25.7 ± 5.5% (*n* = 8) in the presence of control antisense oligonucleotides (Figure 6(c)). In the presence of antisense oligonucleotide against PLC*β*1, cholesterol increased the secreted A*β*42 level only by 2.0 ± 2.1% (*n* = 8). Thus, PLC*β*1 antisense oligonucleotide almost completely prevented the effect of cholesterol on the secreted A*β*42 levels. In contrast, the presence of PLC*β*3 antisense oligonucleotides failed to prevent the effect of cholesterol. The A*β*42 production was still increased by 16.7 ± 3.4% (Figure 6(d); *n* = 6). Together, these results strongly suggest that increased membrane cholesterol levels increased the levels of secreted A*β*42 by down-regulating PIP_2_ levels via specific enhancement of PLC*β*1 expression.

## 4. Discussion

We have previously reported that FAD-linked PS mutants down-regulate PIP_2_ levels, which are closely related to increased A*β*42 level [22]. In addition, up, or downregulation of PIP_2_ levels by pharmacological means decreases or increases the production of A*β*42, respectively. In this study, we showed that cholesterol enrichment increases the secreted A*β*42 levels by down-regulating PIP_2_ levels. Thus, there exists a close relationship between PIP_2_ levels and A*β*42 levels, consistent with our previous results. Recently, we demonstrated that enrichment of cholesterol decreases the levels of PIP_2_ via the activation PLC [23]. Therefore, it was suggested that there exists a crosstalk between two plasma membrane-enriched lipids, cholesterol, and PIP_2_. In this paper, we confirmed that cholesterol decreases the level of PIP_2_ via the activation PLC. In addition, we showed that cholesterol specifically increases the expression levels of PLC*β*1 and PLC*β*3. Consistent with this conclusion, inhibiting transcription prevented the effects of cholesterol not only on PIP_2_ levels but also on the production of A*β*42. Also, the inhibition of PLC*β*1 expression, but not that of PLC*β*3, prevented the effects of cholesterol, indicating a close link between PLC*β*1 and regulation of PIP_2_ levels. 

Although PIP_2_ is a minor component in the plasma membrane, it plays important regulatory roles in a variety of cell functions, such as rearrangement of the cytoskeleton and membrane trafficking [21]. The concept of spatially confined PIP_2_ pools was proposed to explain the multiple roles of PIP_2_ [29]. Cholesterol- and sphingolipid-rich rafts may serve to confine PIP_2_ within the plasma membrane, allowing PIP_2_ hydrolysis to occur locally and restrict signaling mechanisms to the site of activation [30, 31]. In addition to this confined regulation of PIP_2_, it is possible that the steady-state level of PIP_2_ is dynamically determined by the concerted action of phosphoinositide kinases and phosphatases. In this study, we showed another way of regulating PIP_2_ levels within specific microdomains: cholesterol content in a specific microdomain may regulate PIP_2_ levels via PLC activity. Interestingly, PLC*β*1 is shown to localize in detergent-resistant membrane microdomains prepared from the synaptic plasma membrane fraction of rat brain [32]. For this reason, increase of PLC*β*1 expression by cholesterol enrichment may directly induce downregulation of PIP_2_ in the confined microdomain.

The effect of cholesterol enrichment on A*β* secretion was recently demonstrated [33]. It was shown that cholesterol increases clathrin-dependent APP endocytosis, and that it is likely the direct cause of the increased A*β*42 secretion. Since PIP_2_ is a key regulator for the rearrangement of the cytoskeleton and membrane trafficking [21], it is possible that the downregulation of PIP_2_ may be the underlying mechanism for the increased clathrin-dependent APP endocytosis by cholesterol enrichment. Alternatively, the downregulation of PIP_2_ may directly activate *γ*-secretase since PIP_2_ is shown to inhibit *γ*-secretase activity by suppressing its association with the substrate [13]. It is also possible that PIP_2_ induces changes in *γ*-secretase conformation to alter the generation of A*β*42. Recent reports show that the changes in PS1 conformation by various manipulations of PS1 itself, Pen2, Aph1, APP, and pharmacological agents known as *γ*-secretase modulators can allosterically modify *γ*-secretase catalytic specificity, leading to increase of A*β*42/A*β*40 ratio [34, 35]. Further studies will be needed to clarify the role of PIP_2_ in the production of A*β*.

 The physiological relevance of our finding is not clear since a large increase of cholesterol in the membrane was required to induce meaningful downregulation of PIP_2_ and up-regulation of A*β*42 in HeLa cells (Figures 1(c) and 1(e)). In SH-SY5Y cells, however, A*β*42 level was significantly increased even by 15 *μ*M water-soluble cholesterol (Figure 1(f)). We also observed a significant increase of PLC*β*1 expression and downregulation of PIP_2_ level by 15 *μ*M water-soluble cholesterol when we used HEK cells (data not shown). Thus, it seems that the effective amount of cholesterol to induce changes in PIP_2_ and A*β*42 levels is cell type dependent. However, even the minimal increase of A*β*42 by the mild cholesterol enrichment will have profound cytotoxic effects since the cytotoxicity of A*β*42 is caused by its small molecular aggregates, such as dimer form of A*β*42 [9], and the produced A*β*42 will accumulate. It has been demonstrated that cholesterol levels in the brains of AD patients are increased [36, 37]. The levels of cholesterol increase in the brain even during normal aging [36]. In an AD brain, cholesterol homeostasis is impaired, and cholesterol retention likely enhances A*β* production [37]. These results may suggest that high cholesterol levels in the brain participate in the etiology of AD by increasing the generation of A*β*. However, further work is needed to understand how cholesterol is implicated in AD pathogenesis. In this study, we showed that membrane cholesterol levels may share the same molecular mechanism with FAD PS mutations, that is, downregulation of PIP_2_, for the increased generation of A*β*42. Therefore, PIP_2_ may serve as the molecule linking cholesterol metabolism to the pathogenesis of AD.

## Supplementary Material

Supplementary Figure 1: Water-soluble cholesterol increases membrane cholesterol levels.
Supplementary Figure 2: The expression level of PLC*β*1 was increased by cholesterol augmentation.
Supplementary Figure 3: Augmentation of membrane cholesterol levels did not increase the expression levels of PLC*β*2, PLC*β*4, and PLC*γ*1 from APP-transfected HeLa cells.

## References

[1] Hardy J. A., Higgins G. A. (1992). Alzheimer's disease: the amyloid cascade hypothesis. *Science*.

[2] Hardy J., Selkoe D. J. (2002). The amyloid hypothesis of Alzheimer's disease: progress and problems on the road to therapeutics. *Science*.

[3] Walsh D. M., Selkoe D. J. (2007). A*β* oligomers—a decade of discovery. *Journal of Neurochemistry*.

[4] Iwatsubo T., Odaka A., Suzuki N., Mizusawa H., Nukina N., Ihara Y. (1994). Visualization of A*β*42(43) and A*β*40 in senile plaques with end-specific A*β* monoclonals: Evidence that an initially deposited species is A*β*42(43). *Neuron*.

[5] Scheuner D., Eckman C., Jensen M. (1996). Secreted amyloid *β*-protein similar to that in the senile plaques of Alzheimer's disease is increased in vivo by the presenilin 1 and 2 and APP mutations linked to familial Alzheimer's disease. *Nature Medicine*.

[6] De Strooper B. (2003). Aph-1, Pen-2, and nicastrin with presenilin generate an active *γ*-secretase complex. *Neuron*.

[7] Wolfe M. S. (2006). The *γ*-secretase complex: membrane-embedded proteolytic ensemble. *Biochemistry*.

[8] Bentahir M., Nyabi O., Verhamme J. (2006). Presenilin clinical mutations can affect *γ*-secretase activity by different mechanisms. *Journal of Neurochemistry*.

[9] Di Paolo G., Kim T. W. (2011). Erratum: linking lipids to Alzheimer's disease: cholesterol and beyond. *Nature Reviews Neuroscience*.

[10] Refolo L. M., Pappolla M. A., Malester B. (2000). Hypercholesterolemia accelerates the Alzheimer's amyloid pathology in a transgenic mouse model. *Neurobiology of Disease*.

[11] Kalvodova L., Kahya N., Schwille P. (2005). Lipids as modulators of proteolytic activity of BACE: involvement of cholesterol, glycosphingolipids, and anionic phospholipids in vitro. *Journal of Biological Chemistry*.

[12] Osenkowski P., Ye W., Wang R., Wolfe M. S., Selkoe D. J. (2008). Direct and potent regulation of *γ*-secretase by its lipid microenvironment. *Journal of Biological Chemistry*.

[13] Osawa S., Funamoto S., Nobuhara M. (2008). Phosphoinositides suppress *γ*-secretase in both the detergent-soluble and -insoluble states. *Journal of Biological Chemistry*.

[14] Vetrivel K. S., Thinakaran G. (2010). Membrane rafts in Alzheimer's disease *β*-amyloid production. *Biochimica et Biophysica Acta*.

[15] Riddell D. R., Christie G., Hussain I., Dingwall C. (2001). Compartmentalization of *β*-secretase (Asp2) into low-buoyant density, noncaveolar lipid rafts. *Current Biology*.

[16] Ehehalt R., Keller P., Haass C., Thiele C., Simons K. (2003). Amyloidogenic processing of the Alzheimer *β*-amyloid precursor protein depends on lipid rafts. *Journal of Cell Biology*.

[17] Hattori C., Asai M., Onishi H. (2006). BACE1 interacts with lipid raft proteins. *Journal of Neuroscience Research*.

[18] Marquer C., Devauges V., Cossec J. C. (2011). Local cholesterol increase triggers amyloid precursor protein-bace1 clustering in lipid rafts and rapid endocytosis. *FASEB Journal*.

[19] Abad-Rodriguez J., Ledesma M. D., Craessaerts K. (2004). Neuronal membrane cholesterol loss enhances amyloid peptide generation. *Journal of Cell Biology*.

[20] McLaughlin S., Wang J., Gambhir A., Murray D. (2002). PIP2 and proteins: interactions, organization, and information flow. *Annual Review of Biophysics and Biomolecular Structure*.

[21] Di Paolo G., De Camilli P. (2006). Phosphoinositides in cell regulation and membrane dynamics. *Nature*.

[22] Landman N., Jeong S. Y., Shin S. Y. (2006). Presenilin mutations linked to familial Alzheimer's disease cause an imbalance in phosphatidylinositol 4,5-bisphosphate metabolism. *Proceedings of the National Academy of Sciences of the United States of America*.

[23] Chun Y. S., Shin S., Kim Y. (2010). Cholesterol modulates ion channels via down-regulation of phosphatidylinositol 4,5-bisphosphate. *Journal of Neurochemistry*.

[24] Christian A. E., Haynes M. P., Phillips M. C., Rothblat G. H. (1997). Use of cyclodextrins for manipulating cellular cholesterol content. *Journal of Lipid Research*.

[25] Romanenko V. G., Rothblat G. H., Levitan I. (2002). Modulation of endothelial inward-rectifier K^+^ current by optical isomers of cholesterol. *Biophysical Journal*.

[26] Toselli M., Biella G., Taglietti V., Cazzaniga E., Parenti M. (2005). Caveolin-1 expression and membrane cholesterol content modulate N-type calcium channel activity in NG108-15 cells. *Biophysical Journal*.

[27] Ozaki S., DeWald D. B., Shope J. C., Chen J., Prestwich G. D. (2000). Intracellular delivery of phosphoinositides and inositol phosphates using polyamine carriers. *Proceedings of the National Academy of Sciences of the United States of America*.

[28] Zidovetzki R., Levitan I. (2007). Use of cyclodextrins to manipulate plasma membrane cholesterol content: evidence, misconceptions and control strategies. *Biochimica et Biophysica Acta*.

[29] Janmey P. A., Lindberg U. (2004). Cytoskeletal regulation: rich in lipids. *Nature Reviews Molecular Cell Biology*.

[30] Pike L. J., Miller J. M. (1998). Cholesterol depletion delocalizes phosphatidylinositol bisphosphate and inhibits hormone-stimulated phosphatidylinositol turnover. *Journal of Biological Chemistry*.

[31] Hur E. M., Park Y. S., Lee B. D. (2004). Sensitization of epidermal growth factor-induced signaling by bradykinin is mediated by c-Src: implications for a role of lipid microdomains. *Journal of Biological Chemistry*.

[32] Taguchi K., Kumanogoh H., Nakamura S., Maekawa S. (2007). Localization of phospholipase C*β*1 on the detergent-resistant membrane microdomain prepared from the synaptic plasma membrane fraction of rat brain. *Journal of Neuroscience Research*.

[33] Cossec J. C., Simon A., Marquer C. (2010). Clathrin-dependent APP endocytosis and A*β* secretion are highly sensitive to the level of plasma membrane cholesterol. *Biochimica et Biophysica Acta*.

[34] Uemura K., Lill C. M., Li X. (2009). Allosteric modulation of PS1/*γ*-secretase conformation correlates with amyloid *β*42/40 ratio. *PLoS ONE*.

[35] Ebke A., Luebbers T., Fukumori A. (2011). Novel *γ*-secretase enzyme modulators directly target presenilin protein. *Journal of Biological Chemistry*.

[36] Cutler R. G., Kelly J., Storie K. (2004). Involvement of oxidative stress-induced abnormalities in ceramide and cholesterol metabolism in brain aging and Alzheimer's disease. *Proceedings of the National Academy of Sciences of the United States of America*.

[37] Xiong H., Callaghan D., Jones A. (2008). Cholesterol retention in Alzheimer's brain is responsible for high *β*- and *γ*-secretase activities and A*β* production. *Neurobiology of Disease*.

