# Supplementary material for: Increasing Membrane Cholesterol Level Increases the Amyloidogenic Peptide by Enhancing the Expression of Phospholipase C
Source: J Neurodegener Dis. 2013 Mar 7;2013:407903. doi: 10.1155/2013/407903 (PMC4437359; doi:10.1155/2013/407903)
Supplement: Supplementary file 1 — Supplementary Figure 1: Water-soluble cholesterol increases membrane cholesterol levels. Supplementary Figure 2: The expression level of PLCβ1 was increased by cholesterol augmentation. Supplementary Figure 3: Augmentation of membrane cholesterol levels did not increase the expression levels of PLCβ2, PLCβ4, and PLCγ1 from APP-transfected HeLa cells. [file 407903.f1.pdf]

Supplemental Fig. 1.

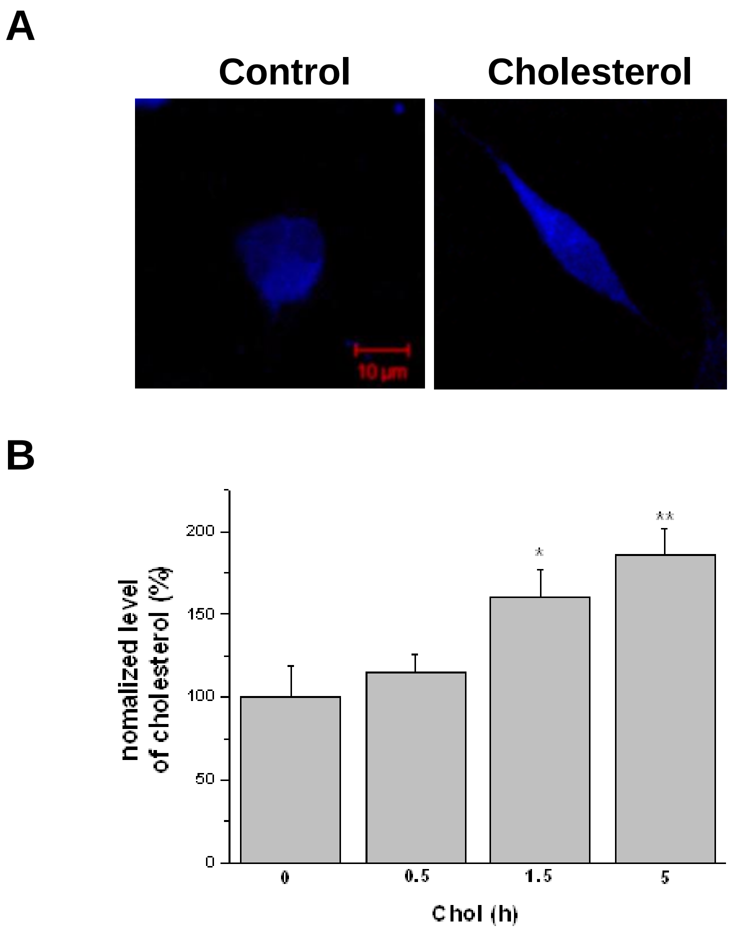

<u>Supplemental Fig. 1.</u> Water-soluble cholesterol increases membrane cholesterol levels. (A) APP-transfected HeLa cells were incubated with 75 µM water-soluble cholesterol for 1.5 h, and filipin staining was performed for 30 min at room temperature. Typical confocal images are shown. (B) The changes of cholesterol levels at the plasma membrane were confirmed by quantifying the fluorescent intensities from plasma membranes after cells were incubated with 75 µM water-soluble cholesterol for 0, 0.5, 1.5, or 5 h (n = 6).

Supplemental Fig. 2.

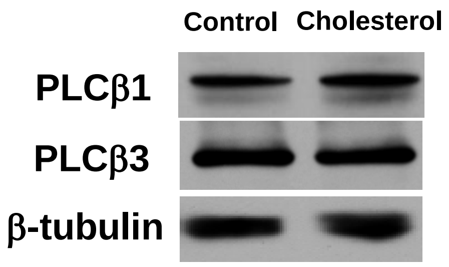

Supplemental Fig. 2. The expression level of PLCβ1 was increased by cholesterol augmentation. To avoid the use of MβCD, membrane cholesterol level was augmented by incubating cells with solubilized cholesterol using sonication. APP-transfected HeLa cells were incubated with 75 μM cholesterol for 1 h, and western blotting from membrane fractions were obtained as described in Materials and Methods. β-tubulin was used to confirm the amount of proteins loaded. Similar results were obtained from 3 different experiments.

Supplemental Fig. 3.

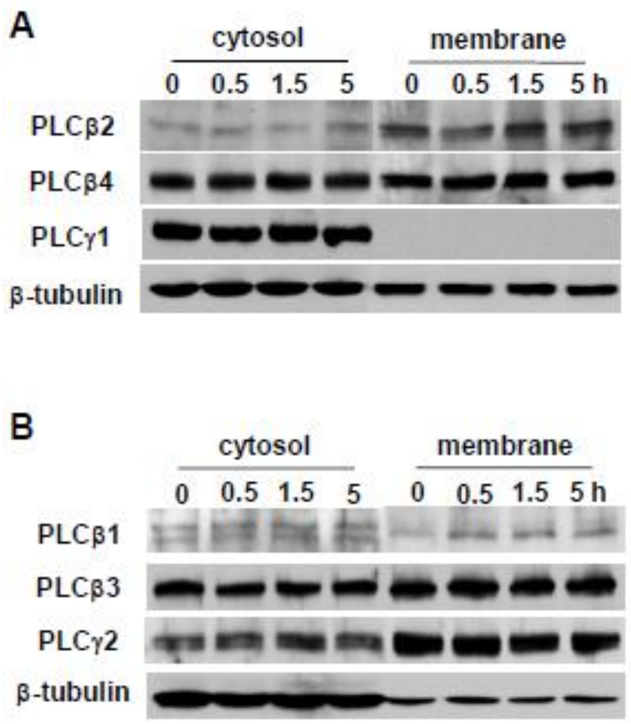

Supplemental Fig. 3. Augmentation of membrane cholesterol levels did not increase the expression levels of PLCβ2, PLCβ4, and PLCγ1 from APP-transfected HeLa cells. (A) Cells were incubated with 75 μM water-soluble cholesterol for the indicated times, and representative Western blotting results were shown. Membrane and cytosol fractions were obtained as described in Materials and Methods. Expression levels of PLCβ2, PLCβ4, and PLCγ1 were not changed by cholesterol. β-tubulin was used to confirm the amount of proteins loaded. Similar results were obtained from 3 different experiments. (B) Representative Western blotting results showed specific increase of PLCβ1 and PLCβ3 expressions from SH-SY5Y cells by augmentation of membrane cholesterol levels. Cells were incubated with 75 μM water-soluble cholesterol for the indicated times. Membrane and cytosol fractions were obtained as described in Materials and Methods. Similar results were obtained from 4 different experiments. Note that PLCβ1 and PLCβ3 expressions increased in time-dependent manner by cholesterol from membrane fraction but not from cytosol fraction. In contrast, PLCγ2 expression was not changed by cholesterol. β-tubulin was used to confirm the amount of proteins loaded.
